# An Inductively Coupled Plasma Source for the Gaseous Electronics Conference RF Reference Cell

**DOI:** 10.6028/jres.100.032

**Published:** 1995

**Authors:** Paul A. Miller, Gregory A. Hebner, Kenneth E. Greenberg, Paul D. Pochan, Ben P. Aragon

**Affiliations:** Sandia National Laboratories, Albuquerque, NM 87185-1423; Department of Chemical and Nuclear Engineering, University of New Mexico, Albuquerque, NM 87131; Applied Physics, Inc., Albuquerque, NM 87110

**Keywords:** GEC RF Reference Cell, high density plasmas, inductively coupled plasmas, Langmuir probes

## Abstract

In order to extend the operating range of the GEC RF Reference Cell, we developed an inductively coupled plasma source that replaced the standard parallel-plate upper-electrode assembly. Voltage and current probes, Langmuir probes, and an 80 GHz interferometer provided information on plasmas formed in argon, chlorine, and nitrogen at pressures from 0.1 Pa to 3 Pa. For powers deposited in the plasma from 20 W to 300 W, the source produced peak electron densities between 10^10^/cm^3^ and 10^12^/cm^3^ and electron temperatures near 4 eV. The electron density peaked on axis with typical full-width at half maximum of 7 cm to 9 cm. Discharges in chlorine and nitrogen had bimodal operation that was clearly evident from optical emission intensity. A dim mode occurred at low power and a bright mode at high power. The transition between modes had hysteresis. After many hours of high-power operation, films formed on electrodes and walls of one Cell. These deposits affected the dim-to-bright mode transition, and also apparently caused generation of hot electrons and increased the plasma potential.

## 1. Introduction

In order to extend the range of operation of the GEC RF Reference Cell [1], we have developed an inductively coupled source that replaces the standard parallel-plate upper-electrode assembly. This source modification generates plasmas with higher electron densities and lower pressures (e.g., 10^12^/cm^3^ at 300 W and 1 Pa) than are obtainable with the original parallel-plate version of the Cell. The new operating regime is more relevant to new generations of industrial plasma tools being developed by the microelectronics industry. At present, three Reference Cells at Sandia are operational with the inductively coupled source and three other laboratories are preparing to operate similar sources in Reference Cells.

Parts for the initial inductively coupled sources were fabricated in 1993 by a local machine shop from sketches produced at Sandia. More recently, a vacuum-component manufacturer has offered a set of parts based on updated Sandia sketches. In order to minimize rf power loss, a custom-built manually tuned impedance-matching network consisting of two air-dielectric variable capacitors was installed immediately on top of the Reference Cell. The total cost of the source, including matching network but excluding rf generator, depends on component selection and on procurement means, but is less than $5000.

As with the original Cell configuration, we intended the source to have satisfactory technical performance, to be inexpensive, and to have excellent diagnostic access. We feel that the present inductively coupled source meets those goals and opens new areas for further studies in discharge operation. The source hardware is described in Sec. 2 and our techniques for implementing Langmuir probes are described in Sec. 3. Section 4 presents argon data and Sec. 5 presents chlorine and nitrogen data. Effects due to films deposited on the electrodes are discussed in Sec. 6.

## 2. Source Configuration and Features

**Fig. 1:**
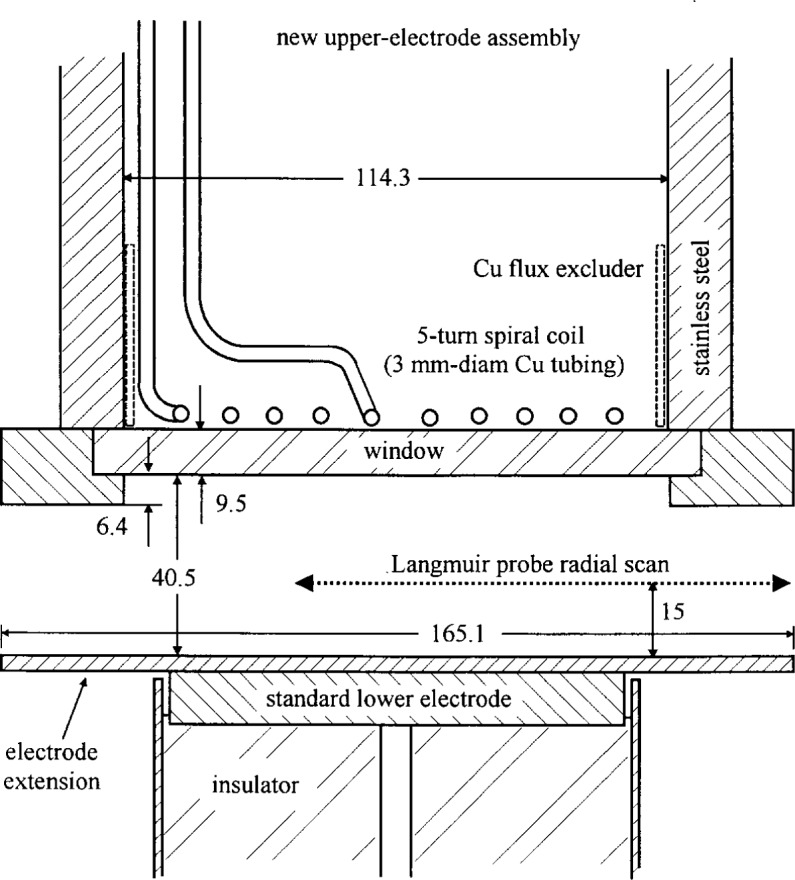
Inductively coupled plasma source with important dimensions in millimeters. The standard body of the Reference Cell (248 mm ID) surrounded this hardware. Gas was injected and evacuated through side ports. The flux excluder (dashed) reduced resistive losses moderately.

The electrode region of the inductively coupled source [2] is shown in Fig. 1. The antenna is a five-turn planar coil of 3 mm (^1^/_8_ in) diameter copper refrigerator tubing (0.75 mm thick walls) that couples to the plasma through a silica window. The top assembly of the source installs in a modified 33.65 mm (13^1^/_4_ in) flange that mates to the Reference-Cell chamber. The source replaces the standard upper electrode assembly. The lower-electrode extension is a disk that rests on top of the standard lower electrode. Bias (dc or rf) can be applied to the lower electrode through the connections that are normally employed to power the standard parallel-plate version of the Cell. A spacer ring in the upper-electrode assembly sets the gap between upper and lower electrodes. The antenna-coupling window provides axial optical access to the plasma. Radial diagnostic access is similar to that of the original parallel-plate version of the Cell. The radial dimensions of the source were chosen as a compromise: plasma modelers wished to have electrodes with infinite radial extent (or a nearby solid cylindrical wall), and experimenters wanted large openings for probes and microwave beams. In the present work, the extent of the plasma beyond the 16.5 cm diameter electrode assembly, which was an initial concern, was found to be acceptably small.

Data were taken using three different inductively coupled sources on three different Cells. As discussed below, the main performance differences among Cells were due to different histories leading to different levels of electrode contamination. The data reported are for the nominally clean condition of electrodes. Except as noted below, data were taken with the center turn of the antenna coil at high voltage and the outer turn grounded. Operation in the opposite configuration was generally similar. As mentioned below, the rf oscillations in plasma potential were small and we have not investigated use of an electrostatic shield between the coil and plasma.

Gas was injected through one side port and exhausted by a throttled turbomolecular pump connected to another side port. Because the pressures were low and the pumping rates were low, the asymmetry of the gas injection and exhaust was not thought to be a significant issue. Flow rates were 3.7 µmol/s to 14.9 µmol/s (5 sccm to 20 sccm) and no flow-rate dependence of results was noted.

A capacitively coupled voltage probe and an inductively coupled current probe were used to measure voltage *V* across, and current *I* through the antenna coil. Construction and operation of probes were as described in Ref. [1]. Antennas used to date have been wound by hand with outside diameter of approximately 10 cm and with approximately equal spacing between turns. Vector-impedance-meter measurements of several antennas gave inductance values *L*=1.2 µH±10 %. The capacitive voltage probe was calibrated *in situ* (without plasma) with reference to a commercial resistive voltage probe. The inductive current probe was subsequently calibrated (without plasma) using the measured *L* and equating the current to *V*/*ωL*, where *ω* is the radian frequency of operation (*ω*=2π×13.56 MHz).

While the source has been operated with a commercial impedance-matching network (pi-network configuration) delivering rf power via coaxial cable, better performance resulted from incorporating a manually tuned capacitive matching network located directly on top of the Cell. The source relies on large circulating rf current (to 25 A) in the antenna. Resistive losses can be large if inductors are used in the matching network or if cables are used to connect the network to the coil. Consequently, the network should avoid use of inductors and output cables. Our matching network employed two air-dielectric variable capacitors with plate spacing of 2 mm. These were suitable for operation at our altitude (1600 m) at antenna voltages above 2 kV. However, for some discharge conditions and gases, high-power operation resulted in excessive voltage across the capacitors which caused breakdown between capacitor plates. There are several considerations and options in design and operation of the matching network that will be the subject of a separate publication. We are developing an automated matching network that uses computer-controlled stepping motors to adjust vacuum-insulated variable capacitors.

A watt meter was used at the input to the matching network and the network was adjusted to minimize reflected power. Reflected power was kept below 1 % of incident power. We present data as functions of “plasma power”. Plasma power refers to the total input power minus *I*^2^*R*_eff_ losses, where *R*_eff_ (typically 0.6 Ω) was due to resistance in the antenna and associated hardware. *R*_eff_ was determined by equating the measured input power to *I*^2^*R*_eff_ during matched-impedance operation under high-vacuum conditions without plasma present. Chilled water flowing through the hollow antenna prevented overheating of the antenna during extended operation in this configuration. Water cooling was used in all our operations, though it may not be needed in many cases. The resistive losses were due in part to currents induced in the stainless-steel cylinder (which became hot to the touch) that surrounded the cool antenna coil. In one test, a copper sleeve was placed between the antenna and the surrounding cylinder in the location indicated in Fig. 1 by the dashed rectangle. The sleeve reduced *R*_eff_ from 0.57 Ω to 0.49 Ω. We expect that significant resistive losses also arose due to currents induced in the electrode extension of the lower electrode, though we did not attempt to measure those losses. Consequently, the resistive losses could be reduced by use of aluminum in the upper and lower electrode assemblies rather than stainless steel.

Accounting for *R*_eff_ caused some uncertainty in our computation of plasma power. If the plasma significantly altered the current distribution in the stainless steel from that occurring in the vacuum case, then *R*_eff_ would change in the presence of plasma and our computed losses (typically 20 % to 30 % of input power) would change. Further work would be needed to account more accurately for resistive losses.

**Fig. 2:**
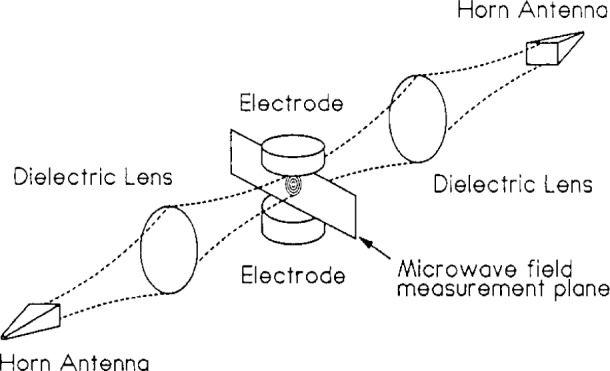
Focusing of the 80 GHz microwave-interferometer beam through large side ports to measure line-integrated electron density. The beam diameter was approximately 1 cm in the interelectrode region.

Figure 1 shows the traversal path for radial scans of a tuned Langmuir probe. That path was chosen to be coincident with the axis of the microwave beam from an 80 GHz interferometer that was used for electron density measurements. The Langmuir probe work is described below. The interferometer, which was described in detail in Ref. [3], employed dielectric lenses as shown in Fig. 2 to form a microwave beam that passed through large ports on opposite sides of the Cell chamber and midway between the upper and lower electrodes. The beam diameter was 1 cm in the electrode region. A line-integrated electron density of 1.7×10^11^/cm^2^ gave a phase shift of 1°, which was approximately the minimum detectable phase shift for the interferometer.

## 3. Probe Techniques

### 3.1 Langmuir Probe

**Fig. 3:**
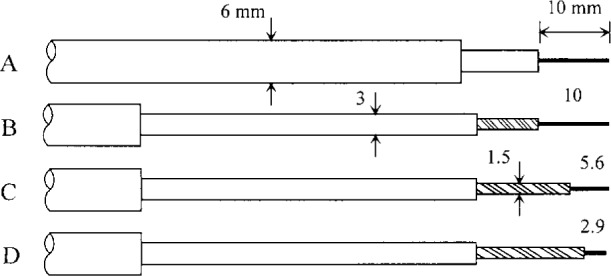
Four different Langmuir probes for measuring eedfs with important dimensions in millimeters. The tips all were 0.63 mm diameter wire. The 1.5 mm diameter insulator was alumina; the larger insulators were Pyrex.

Langmuir probe measurements can be misinterpreted easily and a description of our approach is warranted. In general, we heeded the admonitions of Godyak [4]. For our work, the regime of interest was densities of 10^10^ electrons/cm^3^ to 10^12^ electrons/cm^3^, effective bulk electron temperatures of 1 eV to 10 eV, and gas pressures of 0.5 Pa to 3 Pa (4 mTorr to 23 mTorr). The corresponding range of Debye lengths is 0.01 mm to 0.15 mm. The probe electrical element was 0.63 mm diameter nickel-chrome wire. This diameter was chosen to be much larger than the typical Debye length of interest so that probe-area corrections could be neglected. The probe-wire material did not appear to interact chemically with any of the discharge-gas species. Four different geometries of probes were used. They are shown approximately to scale in Fig. 3. The succession of smaller probe tips and tip holders were tested to measure the extent to which the probes perturbed the plasma. Probe A, the largest, had a wire surface area of 0.2 cm^2^. Geometric shadowing of the wire by the 3 mm diameter Pyrex wire holder reduced the solid angle available for collection, and the effective area, by 7 %. This wire holder was in turn supported by a 6 mm diameter Pyrex tube that provided the main mechanical support for the 60 cm long probe assembly. The probe assembly was attached to a manually operated *x*-*y*-*z* translation stage which was attached to one of the 6.98 mm (2^3^/_4_ in) flanges on the Reference Cell. The stage had a 15 cm translation capability along its *z* axis. The probe-wire axis was oriented radially in the Cell, parallel to the lower electrode’s surface. Probes B, C, and D were supported in the same manner as was probe A. Data comparing the results from the probes are presented below.

For the plasma parameters of interest, mean free paths were much larger than the probe diameter and the Debye length was much less than the probe diameter, and, consequently, simple one-dimensional probe theory was deemed adequate. However, axial gradient scale lengths in the plasma were approximately 1 cm which was less than typical mean free paths. Consequently, distribution functions could have been anisotropic, leading to uncertainty in interpretation of probe data.

The probe was biased negatively 20 V to 40 V with respect to the plasma potential to maintain constant probe surface conditions. The probe was pulsed positive by triangular pulses with duration ≤1 ms at a rate of 10 Hz to 20 Hz. We found, as reported by Godyak [4], that the probe curves displayed hysteresis when pulse durations of 20 ms to 50 ms were used. This implied the presence of changing probe surface conditions during those slower pulses.

**Fig. 4:**
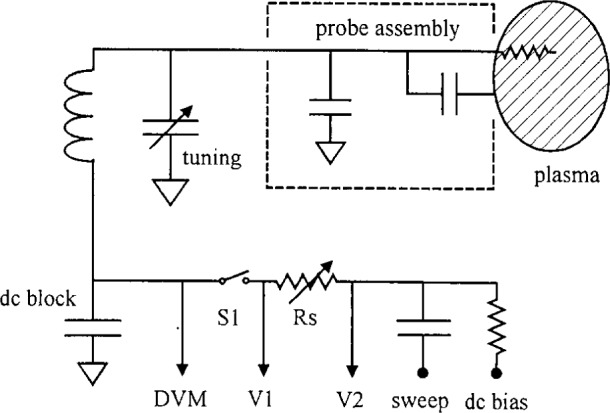
Probe-bias circuit. When the resonant circuit was being tuned, switch S1 was set to the open position and a digital voltmeter (DVM) was used to indicate floating potential, which was maximized by adjusting the variable capacitor. During probe measurements, V1 indicated probe voltage and (V2-V1)/Rs indicated probe current. The value of Rs was increased for measurements of low currents (low electron densities).

The probe’s bias circuit included a tunable *L*-*C* shunt circuit [5] that resonated at the plasma-excitation frequency (13.56 MHz) to allow the probe to follow the high-frequency oscillations of the plasma potential and thereby to reduce distortion of current-voltage (*I*–*V*) curves. Figure 4 shows a schematic of the bias circuitry. With switch S1 closed, V1 indicated the probe voltage and the difference between V1 and V2 indicated the probe current. Series resistor Rs (22 Ω to 1000 Ω) was adjusted for optimum signal levels at different plasma densities. The circuit’s resonant frequency was tuned to maximize the measured dc component of the probe’s floating potential, which was measured by a digital voltmeter (DVM) with switch S1 open. At lower plasma densities, the tuning resonance was sharp; at higher densities the resonance was broad. This behavior was expected because the impedance of the probe-to-plasma junction decreased as the sheath thickness decreased (with increasing plasma density). As a numerical example, a 4 eV plasma with 8.8×10^10^/cm^3^ electron density has a Debye length of 0.05 mm. With probe A (0.2 cm^2^ area), the probe-to-plasma vacuum capacitance across such a sheath is calculated to be 3.5 pF which has a reactance at 13.56 MHz of 3.3 kΩ, whereas the impedance of the *L*-*C* circuit was measured using a vector impedance meter to be 31 kΩ at resonance. Thus the effect of the plasma-potential oscillations at 13.56 MHz on the *I*–*V* curves would be reduced by approximately a factor of 10 (=34.3/3.3) for this example. This estimate is conservative, however, because it neglects the effect of conduction current in the sheath on reducing the probe-to-plasma sheath impedance. Probe data showed that, for a case closely matching this numerical example, the differential impedance of the *I*–*V* characteristic was actually between 60 Ω and 500 Ω for more than 90 % of the probe sweep. Consequently, the effect on probe A of the plasma potential oscillations at 13.56 MHz would be reduced by a factor exceeding 50. Measurement of the plasma oscillations is described in Sec. 3.2.

The probe electrical data (V1 and V2) were recorded using a two-channel digital oscilloscope. The oscilloscope technique was very convenient for us and provided the necessary high-speed data acquisition to resolve the triangular pulses on a single-shot basis. However, it did not allow resolution of the tail of the electron energy distribution function (eedf) because of the limited resolution and range of the oscilloscope’s 8 bit analog-to-digital converter. This could be improved upon in future work by simultaneously employing two oscilloscopes set at different sensitivities [6] or by using higher-resolution recorders.

The I–V characteristics of the Langmuir probe were derived from the oscilloscope data using an equivalent-circuit model for the bias circuitry that included corrections for parasitic inductance and capacitance which are not indicated in Fig. 4. The model was developed and tested with the probe replaced by resistive loads as well as open- and short-circuit loads. The first and second derivatives of the probe *I*–*V* curve were obtained by fitting cubic polynomials to the data at numerous points and by evaluating the analytic derivatives of the polynomials. The range of fitting was varied from, typically, 1 V near the plasma potential to several volts in the tail of the distribution. This provided the eedf and identified the plasma potential *V*_p_ (d^2^*I*/d*V*^2^=0 and d*I*/d*V* is a maximum at *V*=*V*_p_). The computed second derivative usually peaked at 1 eV to 2 eV below *V*_p_, which is a measure of the validity of our techniques [4]. However, this does not indicate the presence of such an error in the values obtained for *V*_p_. Our use of cubic polynomials limited the accuracy of eedf computations near *V*_p_ because the discontinuity in d^2^*I*/d*V*^2^ was not treated precisely. Further algorithm development probably could reduce the numerical contribution to the energy of the peak. For example, if the cubic-polynomial fit were restricted to a single-sided fit with *V*<*V*_p_, then the fit would have more noise near the peak but the discontinuity could be approached more closely without affecting the fit. Alternatively, one could replace the cubic polynomial form with a more complicated form that included an explicit break at *V*_p_.

Electron density and effective bulk electron temperature were obtained from probe data in two different ways. *First*, a Maxwellian distribution was fitted to the eedf over the range 4 eV to 15 eV (typically) to obtain a temperature and the eedf was integrated directly to obtain an electron density. The derivative signals were frequently noisy and, thus, the fitted temperatures varied erratically by 0.5 eV. *Second*, using the value of *V*_p_ from the differentiated signal, a Maxwellian distribution was fitted to the original *I*–*V* curve in the region *V*<*V*_p_, with a constant displacement allowed for the ion current. Values of electron density and temperature were then calculated from the two free parameters of that fitted Maxwellian.

The first data-analysis technique gave electron densities that were 10 % to 15 % lower than the second technique, presumably because of the error in the eedf below the peak at 1 eV to 2 eV. The electron temperatures obtained by the two techniques had no consistent differences. Consequently, we report below the results of the Maxwellian fits to the original *I*–*V* curves (the “second” technique, which had lower noise), which were obtained using *V*_p_ from the differentiated data. Except as discussed in Sec. 6, the Maxwellian fits to the data were subjectively quite satisfactory.

### 3.2 Capacitive Voltage Probe

The rf components of the oscillating plasma potential were measured directly using a capacitive probe immersed in the center of an argon plasma. The probe sensing element (<1 cm^2^ area) was attached to the end of the center conductor of a semirigid coaxial cable. The sense element and the attached cable were enclosed in 6 mm diameter Pyrex tubing. The cable was terminated in 50 Ω at a digital oscilloscope and the signal was integrated to provide an rf voltage measurement. The probe was calibrated by tightly wrapping the tip of the Pyrex tube with aluminum foil and by exciting the foil with a calibration signal from a signal generator. The relationship of the measured excitation-signal amplitude to the probe response provided a calibration constant.

With the outside of the induction coil powered (center grounded) over the range 30 W to 170 W, the rf components of the plasma potential were found to be 1.5 V to 2 V amplitude at 13.56 MHz, 0.5 V at the second harmonic, and less than 0.08 V at higher harmonics. The harmonic amplitudes *decreased* with increasing power. With the center of the coil powered (outer turn grounded), the oscillating potentials were approximately twice as large as with the outside powered. Because these oscillating potentials were so small, the signal distortion for the tuned Langmuir probe was expected to be fairly small and predominantly due to the second harmonic, except perhaps at the lowest densities. The distortion due to the second harmonic would be reduced if the Langmuir-probe-bias circuit were modified by adding a resonance at the second harmonic. Since, in either configuration of coil excitation, the plasma-potential oscillations were only a few volts, we did not pursue development and testing of an electrostatic shield for the inductively coupled source.

At rf input powers above 170 W, the Pyrex tube surrounding the capacitive-probe tip was heated by the plasma sufficiently to cause expansion and flow of insulating material in the tip and to cause melting of soft solder in the tip [melting point 455 K (360 F)]. This severe thermal environment might prove troublesome for some rf filters that have been used previously in the tips of Langmuir probes by other workers. We avoided the use of solder or adhesives in the tips of our Langmuir probes, which were used at powers to 300 W.

## 4. Argon Discharges

**Fig. 5:**
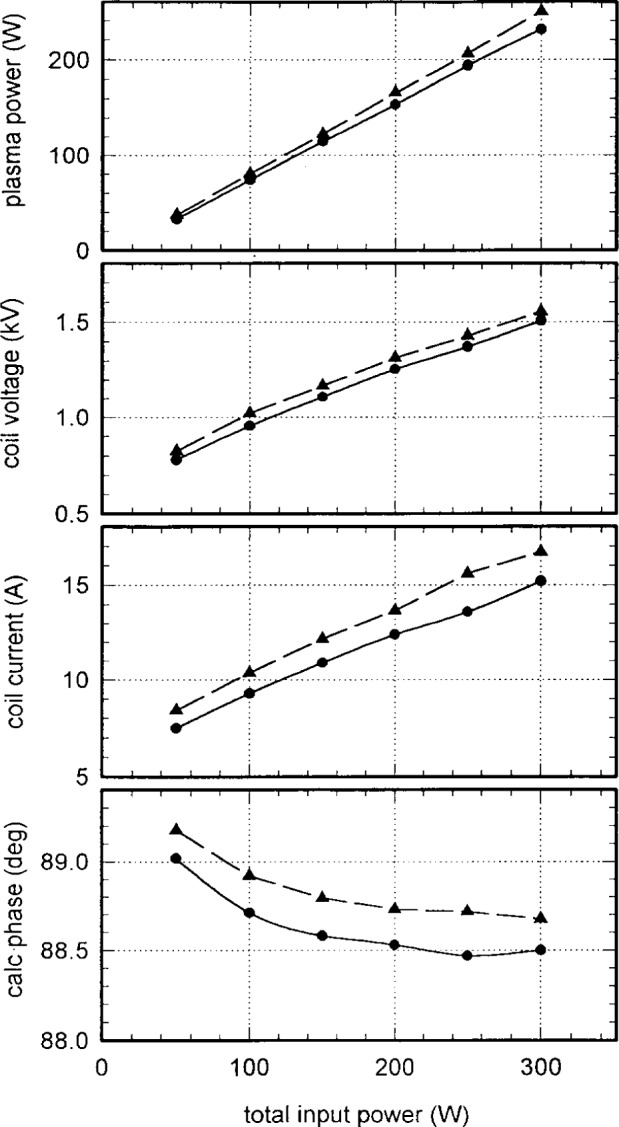
Antenna parameters for 2 Pa (15 mTorr) argon discharges in two different Reference Cells. The upper curves (dashed) are for a source with unusually low *R*_eff_=0.36 Ω. The solid curve is for a source with *R*_eff_=0.52 Ω. The phase was calculated from *V*, *I*, and power data.

Argon discharges were generally very stable. Figure 5 shows voltage and current data at the fundamental frequency for the antenna coil for discharges in 2 Pa (15 mTorr) argon. Harmonics amplitudes were less than 1 % of the fundamental. Values of rf voltage and current are reported in this paper as zero-to-peak values, i.e., one-half the peak-to-peak values. Data are shown for two different Cells which had *R*_eff_=0.36 Ω and 0.52 Ω, and *L*=1.15 µH and 1.22 µH, respectively. This 30 % difference in resistances was the biggest seen throughout several iterations of antennas and Cells and no specific cause was identified for the large variation in this particular case. However, as evident from Fig. 5, the difference in *R*_eff_ did not cause major differences in coil parameters or plasma power. Usually, *R*_eff_ was between 0.5 Ω and 0.7 Ω. The phase angles in Fig. 5 were calculated to make the voltage and current values consistent with the plasma power, as defined in Sec. 2. The phase angles were very close to 90° and, consequently, they could not be measured with sufficient accuracy for reliable power computation by our standard data acquisition techniques [1]. The coupling of the antenna to the plasma was sensitive to any spacing between the antenna and the coupling window. In one test, in order to maintain constant plasma power, the required antenna current increased by 50 % when the antenna was moved approximately 6 mm away from the window.

**Fig. 6:**
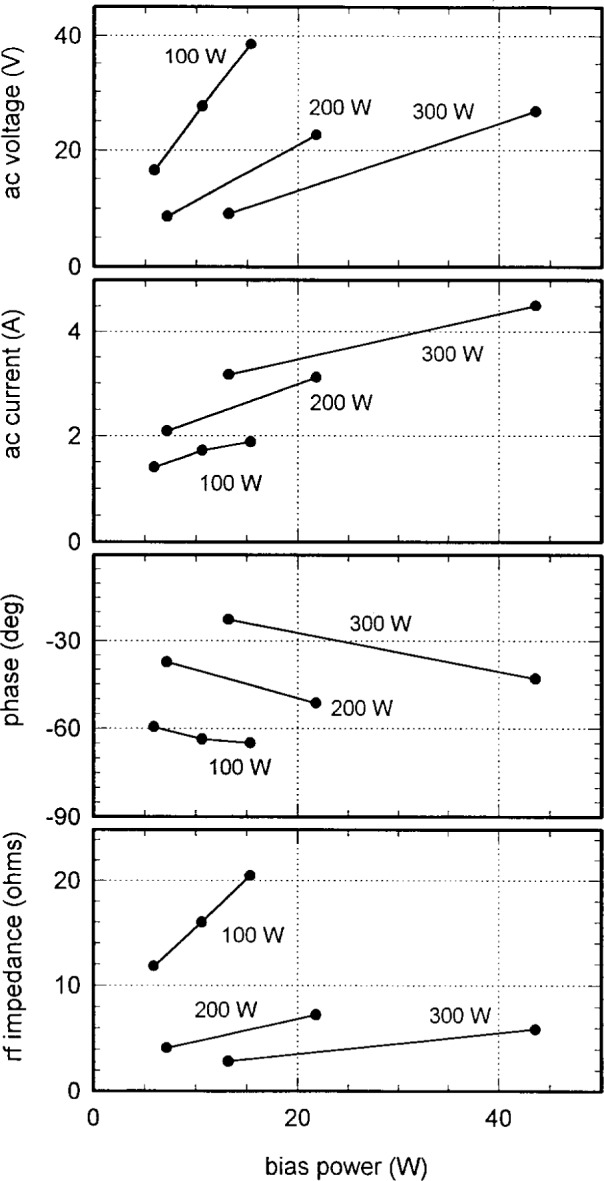
RF parameters of the lower electrode vs applied rf bias for 2 Pa argon discharges. The curves are labeled with rf input power to the coil antenna.

**Fig. 7:**
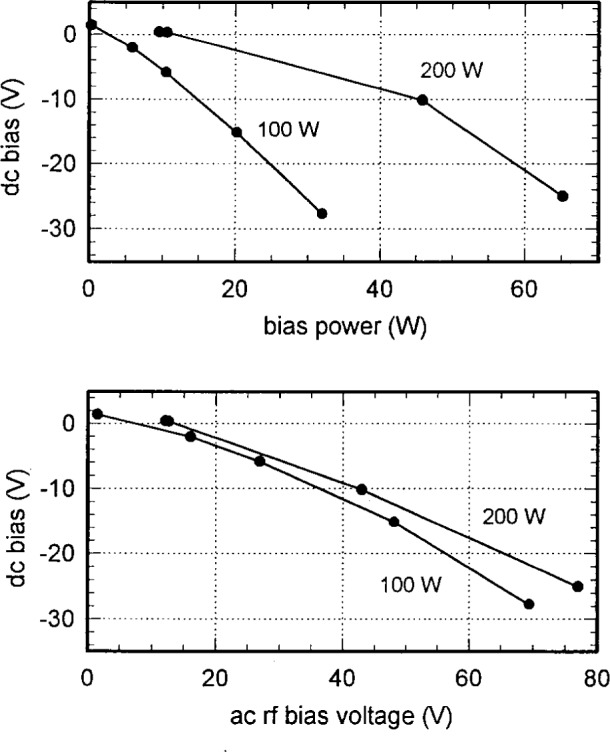
Relation of dc bias on the lower electrode to applied rf power and voltage for 2 Pa argon discharges.

Figures 6 and 7 show voltage and current data at the fundamental frequency for the bias electrode for 2 Pa argon discharges for several rf antenna input powers. Measured *V* and *I* values have been converted to values at the electrode for Figs. 6 and 7 by use of standard capacitive and inductive corrections [1]. The rf bias was provided by a tee connected to the main source of rf power supplying the antenna coil. The bias amplitude was adjusted by varying a series coupling capacitor. Relative phase between the antenna and bias signal was neither determined nor controlled. Figure 6 shows that the electrode-to-plasma impedance decreased in magnitude and became more resistive as bias power increased. Figure 7 shows how the resulting dc bias was related to the rf bias power and voltage. Caution should be exercised in operating with high bias voltage. In one case, extended operation with high bias voltage sputtered enough metal from the bottom electrode onto the coupling window to make it opaque (mirror-like). The metal coating attenuated the power delivered from the antenna coil to the plasma and the window was difficult to clean. All other data reported in this paper were taken with the lower electrode grounded.

**Fig. 8:**
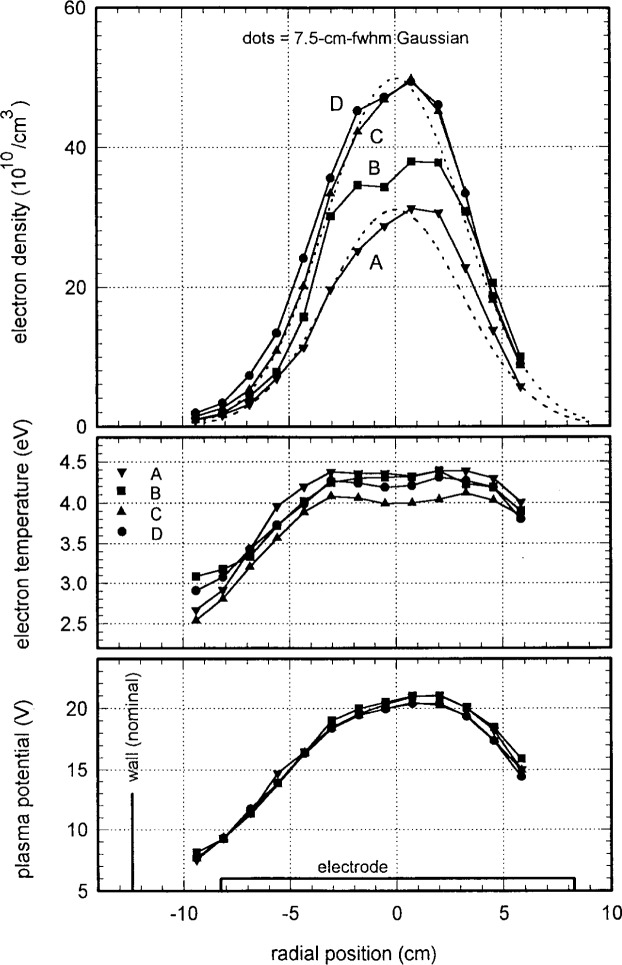
Radial profiles of electron density and temperature and of plasma potential for the four Langmuir probes of Fig. 3. Data are from 1.33 Pa argon discharges with 118 W plasma power. The axial position of the probes was 15 mm above the lower electrode. The radial position of the chamber wall and the extent of the lower electrode are indicted in the bottom graph.

**Fig. 9:**
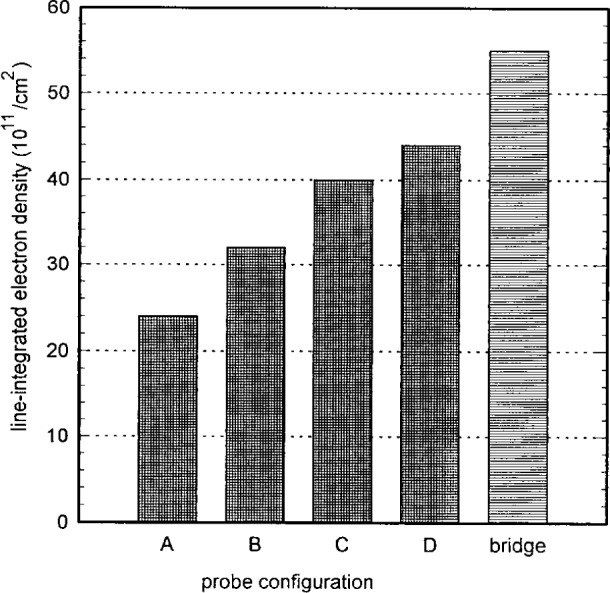
Line-integrals of the data in Fig. 8 compared to microwave bridge data. As the probe size was decreased, the probe values approached the microwave value.

**Fig. 10:**
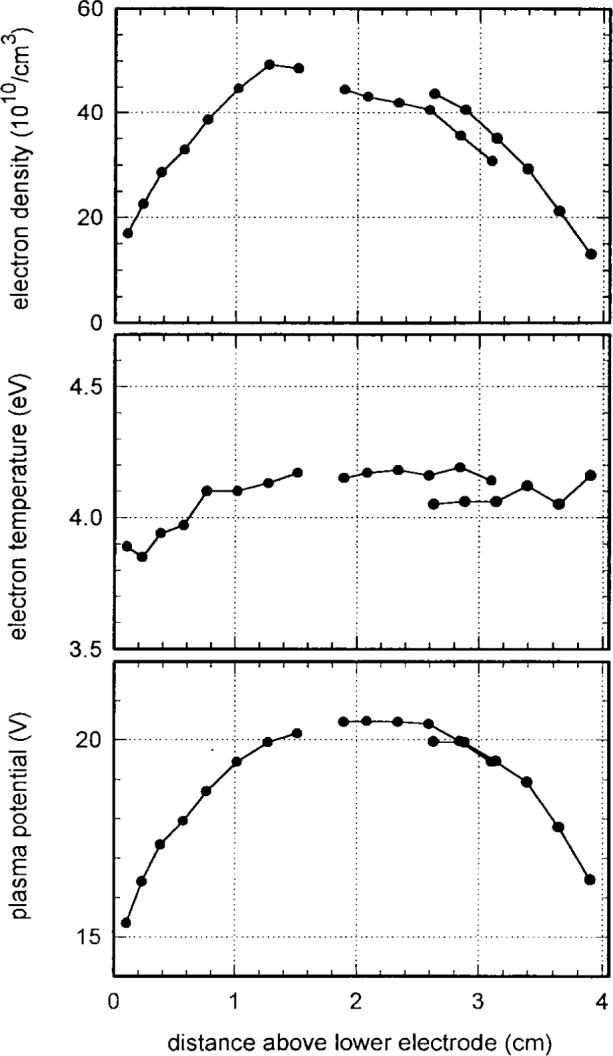
Axial variation of electron density and temperature and of plasma potential. Three different probes, all type D, were used to obtain the axial scan.

**Fig. 11:**
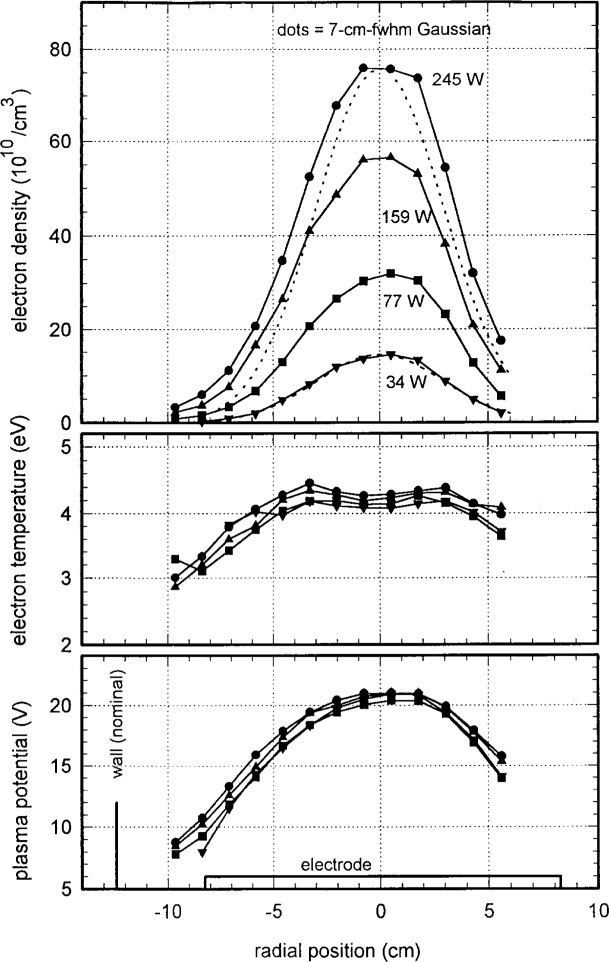
Variation with power of plasma parameters for 1.33 Pa argon discharges. For reference, Gaussian profiles have been matched to the peaks of the 34 W curve and the 245 W curve.

**Fig. 12:**
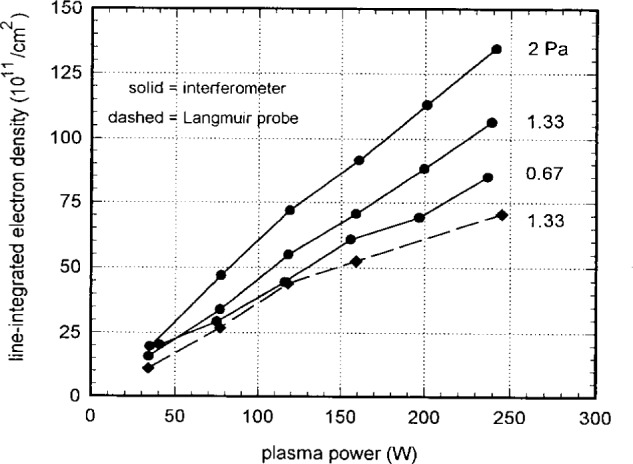
Integrated electron densities for argon discharges. The Langmuir probe data (type-D probe) for 1.33 Pa are approximately 30 % below the corresponding microwave data, as is the case in Fig. 9.

**Fig. 13:**
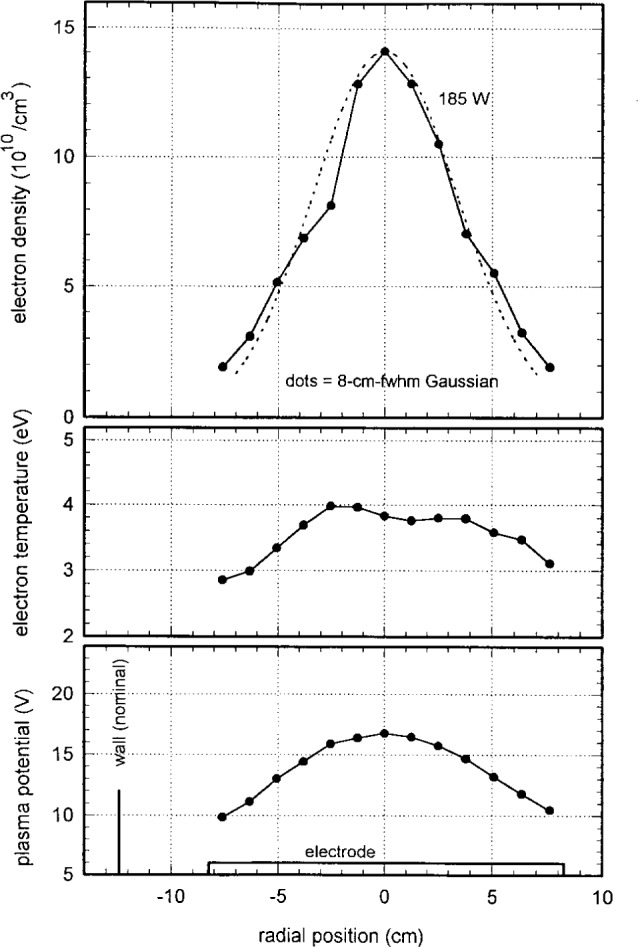
Radial profiles for 185 W discharge in 2.67 Pa chlorine from probe D. Although the chlorine data had more variability than the argon data, the overall shapes were quite similar.

**Fig. 14:**
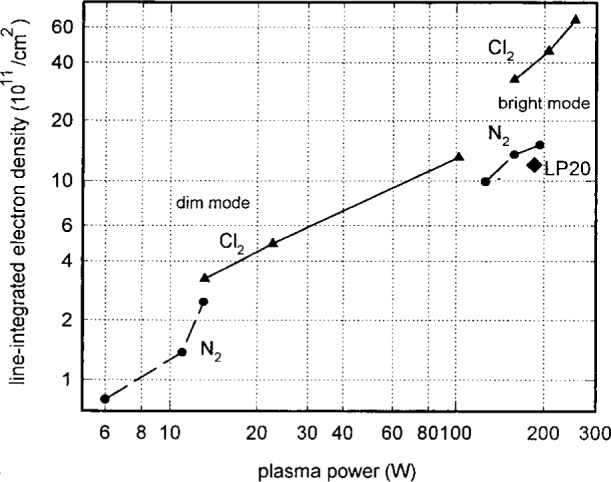
Integrated electron density for 1.33 Pa chlorine and 2.67 Pa nitrogen discharges. Electron density and power jumps were seen for both gases when the discharges switched from the dim to bright mode.

Figure 8 shows results from radial scans of Langmuir probes A, B, C, and D in 1.33 Pa (10 mTorr) argon discharges with 150 W total input power (118 W plasma power) with the probes 15 mm above the lower electrode (see Fig. 1). Refer back to Fig. 3 for probe geometries. As the probe became smaller, the implied electron densities increased, but the temperatures and plasma potentials remained fairly constant. There was no clear reason why the electron temperature from probe C was 0.3 eV lower than from the other probes. The plasma potentials all were similar. Figure 9 shows a comparison of the line-integrated electron densities from the four probes with the value from the microwave interferometer (bridge). We conclude that all the probes perturb the plasma significantly and that probe D gives closest to the correct value for electron density. Subsequent data were taken with probe D, and the electron densities reported in Figs. 10–14 have *not* been adjusted upwards with the correction factor suggested by Fig. 9.

We noted that, at powers of 150 W and higher, strong low-frequency oscillations (e.g. <10 Hz, 10 % amplitude) were present on probe signals when the probe was extended beyond the center axis, deep into the plasma. We did not see (by eye) any flickering of the discharge light. In order to obtain probe traces when oscillations were present, the low-frequency oscillations were simply averaged away using the averaging feature of the digital oscilloscope. We did not observe any degradation in the quality of the analytic fits to the averaged data due to the oscillations. It is surprising that the probe data do not show more spatial asymmetry due to the probe-induced oscillations.

Figure 10 shows an axial scan, on axis at *r*=0, between the lower electrode and the coupling window, for 1.33 Pa argon discharges with 150 W input power. To acquire these data, three versions of probe D were used. Due to restrictions on the transverse travel of the probe stage, and due to obstruction by the clamp ring that holds the coupling window, we had to use probes with bends in the 3 mm diameter Pyrex section near the tip. The disagreements in Fig. 10 at the joining and overlap regions of the three probe curves provide a measure of the reproducibility of our techniques. The disagreement in densities was most probably due to an error in setting probe wire length.

It is interesting to note the difference in the variation of bulk electron temperature in the axial and radial directions. In the axial direction (Fig. 10), the temperature is quite constant over the extent of the plasma. In the radial direction (Fig. 8), the temperature drops smoothly beyond the radius of the edge of the induction coil. In the axial direction the plasma is confined by abrupt sheaths at the electrodes while in the radial direction the plasma can stream away freely from the source region. The electrons are collisional with themselves, but nearly collisionless with the neutrals. The absence of nearby confining walls in the radial direction, which enhances diagnostic access, distinguishes the Reference-Cell geometry from many other inductively coupled sources. We have not evaluated in detail the decrease in electron temperature at large radii, but we speculate that the decrease may be caused by cooling of the electrons due to volumetric expansion. This subjects merits further consideration.

Figure 11 presents radial scans for 1.33 Pa argon discharges at various levels of plasma power. The peak electron density increased with power and the radial profile broadened slightly. The electron temperatures and plasma potentials show no clear variation with power. A separate set of probe scans (not shown) that extended out to the chamber wall showed the continued rapid decay of the plasma with increasing radius. Figure 12 shows a comparison of Langmuir-probe data and interferometer data. Line-integrated values of electron density for the profiles in Figs. 8 and 11 are shown along with interferometer data for 3 different argon pressures. As in Fig. 9, the probe-D values in Fig. 12 are approximately one-third below the interferometer values.

## 5. Chlorine and Nitrogen Discharges

Discharges in all gas species showed bimodal behavior with hysteresis in switching between modes, as has been seen in other discharge systems. At low input power, most of the input power was dissipated by resistive losses (*I*^2^*R*_eff_) and the optical emission was weak. This was termed the “dim” mode. In argon, a mild transition to a brighter mode occurred at low power, and all the data reported above (Figs. 5–12) are for this “bright” mode. In chlorine and nitrogen, the dim mode persisted above 100 W. As input power was increased above 100 W, the discharge often changed suddenly and dramatically, at 150 W to 250 W, to the bright mode in which most of the input power went into the plasma and the optical emission increased manyfold. For example, in one test with 2.67 Pa of nitrogen, the dim mode persisted up to 175 W input power (<1 W reflected), of which 150 W was dissipated resistively and 25 W went into the plasma. The antenna *V* and *I* were 2252 V and 21.47 A, with implied phase angle of 89.59°. The optical signal from a broad-band silicon photodiode viewing the plasma was 0.07 V. Once ignited, the bright mode could be maintained down to 200 W input power (<1 W reflected), of which 77.5 W was dissipated resistively and 122.5 W went into the plasma, a five-fold increase in plasma power over the dim mode. Antenna parameters in this case were 1639 V, 15.44 A, and 89.09°. The optical signal jumped to 1.3 V in the bright mode, an increase of a factor of 18.6. Stable operation was not obtained in that test at input powers between 175 W and 200 W. At times, when the discharge was marginally stable in the bright mode, insertion of the Langmuir probe into the discharge caused a mode change or discharge extinction.

We use the terms “dim” and “bright” to describe the most obvious feature of these modes. It is commonly accepted that these modes are ***E*** and ***H*** modes that are dominated by capacitive and inductive coupling, respectively. The mode change in the molecular gases could also be associated with a change from domination by molecular neutrals and/or ions to atomic species, which would cause a large change in kinetic rates. It should be noted that simple nonlinear differential equations, which could describe the interaction of plasma and the matching network, can give rise to strong bimodal behavior [7]. Phenomenologically similar multi-mode behavior has been reported in parallel-plate systems [8] where ***H*** modes should be absent, and in ECR systems [9]. This subject deserves further study, particularly to elaborate the mechanism controlling the mode-transition point in different geometries and gases.

Figure 13 shows radial profiles obtained with the Langmuir probe in a 2.67 Pa chlorine discharge at plasma power of 185 W. Because the chlorine discharge was less stable than argon discharges and the electron density was lower, these data had higher noise levels. The shapes in Fig. 13 are remarkably similar to those for argon in Fig. 11. The electron density was several times higher in argon than in chlorine at the same power, and the plasma potential was somewhat higher in argon.

Langmuir-probe data for a 2 Pa nitrogen discharge at 167 W plasma power indicated similar radial profiles as for the other gases. The peak electron density was approximately 6×10^10^/cm^3^. The plasma potential was 24 V peak and the peak electron temperature was between 5 eV and 6 eV.

Figure 14 shows microwave interferometer data from 1.33 Pa chlorine discharges and from 2.67 Pa nitrogen discharges in the dim and bright modes. In agreement with the Langmuir probe data, the electron densities in nitrogen discharges were approximately a factor of three lower than in chlorine discharges. It is remarkable that the change in electron density between dim and bright modes was only a factor of three while the change in optical emission was nearly a factor of 20. The one isolated data point in Fig. 14 (labeled LP20) is the integrated value from the Langmuir-probe data in Fig. 13 from a 2.67 Pa chlorine discharge. Because of the pressure difference, that Langmuir probe measurement is not directly comparable with the microwave chlorine measurements in Fig. 14. We did not take Langmuir-probe data in chlorine discharges at 1.33 Pa because, at that point in the experiments, the probe and interferometer were used with different Cells that operated differently for reasons discussed below.

## 6. Electrode-Coating Effects

As mentioned previously, we employed three different Reference Cells with inductively coupled sources in this work. After extensive high-power operation in several gases, one Cell developed visible coatings on the metallic electrode surfaces. The coatings were transparent and showed several cycles of interference fringes when viewed in ambient fluorescent light. Surface analysis by Raman scattering, infrared absorption, and x-ray fluorescence indicated that the film contained predominantly silicon and oxygen. The Si and O appeared to be a form of silica deposited at high temperature, and probably were sputtered from the rf coupling window.

We demonstrated two obvious effects due to the coatings. The cause-and-effect relationships were established by selectively replacing coated electrodes with clean electrodes and by observing resulting changes in Cell operation. *First*, in chlorine discharges, the coated Cell operated stably in the bright mode at 1.33 Pa, whereas a clean Cell did not. For example, in a clean Cell in the dim mode at 1.33 Pa, as rf input power was increased above approximately 130 W, the discharge extinguished completely. In the clean Cell, the pressure had to be above 2 Pa to obtain stable bright-mode operation in chlorine. *Second*, Langmuir probe data from argon discharges in the coated Cell showed elevated plasma potential (to 45 V) and higher effective electron temperatures (10 eV). However, unlike the lower temperatures for clean Cells, these higher “temperatures” actually gave poor fits to the probe data. The *I*–*V* curves and computed eedfs for the coated Cell were clearly non-Maxwellian, with one or two large groups of hot electrons in addition to a low-energy group. It is puzzling, however, that electron density and optical emission were not grossly affected by the changed plasma conditions. Consequently, it is possible that the computed eefds with hot electrons are actually indicative of some unknown effect that affected the validity of the Langmuir-probe data analysis.

If copious hot electrons were actually to occur in a microelectronics production reactor, circuit-damage mechanisms would be affected. The elevated plasma potential would increase ion impact energies. Energetic electrons could cause damage either directly or indirectly by increasing production of ultraviolet radiation. Further work is needed to understand and validate the chemical and/or electrical mechanisms of the interaction of the coating with the discharge.

## 7. Summary

The inductively coupled source developed for the GEC RF Reference Cell successfully generated high-density low-pressure plasmas. Langmuir-probe and microwave diagnostics showed that electron densities approaching 10^12^/cm^3^ were obtained in 1.33 Pa argon at 300 W input. The powers per unit volume and per unit area were similar to those obtained in industrial high-density reactors while the unique geometry of the Reference Cell provided exceptional diagnostic access. While the plasma was not radially confined to the electrode region, only a few percent of the line-integrated electron density extended beyond the edge of the electrodes. Oscillations in plasma potential were a few volts in argon, suggesting that an electrostatic shield between coil and plasma is not needed for many purposes. Discharges in argon were stable, but discharges in chlorine and nitrogen displayed instability and multimode operation as have been reported in other systems. These instabilities compromise plasma control and repeatability. Further study is needed of discharge instabilities, multi-mode operation, and effects of electrode coatings in the Reference Cell.
